# A study protocol for the development of a multivariable model predicting 6- and 12-month mortality for people with dementia living in residential aged care facilities (RACFs) in Australia

**DOI:** 10.1186/s41512-020-00085-0

**Published:** 2020-10-07

**Authors:** Ross Bicknell, Wen Kwang Lim, Andrea B. Maier, Dina LoGiuidice

**Affiliations:** 1 Department of Medicine and Aged Care, @AgeMelbourne, Melbourne Health–Royal Melbourne Hospital, University of Melbourne, 6 North Main Building, Royal Melbourne Hospital, 300 Grattan Street, Parkville, Victoria 3050 Australia; 2 Department of Human Movement Sciences, @AgeAmsterdam, Amsterdam Movement Sciences, Vrije Universiteit Amsterdam, Amsterdam, The Netherlands

**Keywords:** Dementia, Mortality, Prognosis, Predictive modeling, Residential aged care, Long-term care

## Abstract

**Background:**

For residential aged care facility (RACF) residents with dementia, lack of prognostic guidance presents a significant challenge for end of life care planning. In an attempt to address this issue, models have been developed to assess mortality risk for people with advanced dementia, predominantly using long-term care minimum data set (MDS) information from the USA. A limitation of these models is that the information contained within the MDS used for model development was not collected for the purpose of identifying prognostic factors. The models developed using MDS data have had relatively modest ability to discriminate mortality risk and are difficult to apply outside the MDS setting. This study will aim to develop a model to estimate 6- and 12-month mortality risk for people with dementia from prognostic indicators recorded during usual clinical care provided in RACFs in Australia.

**Methods:**

A secondary analysis will be conducted for a cohort of people with dementia from RACFs participating in a cluster-randomized trial of a palliative care education intervention (IMPETUS-D). Ten prognostic indicator variables were identified based on a literature review of clinical features associated with increased mortality for people with dementia living in RACFs. Variables will be extracted from RACF files at baseline and mortality measured at 6 and 12 months after baseline data collection. A multivariable logistic regression model will be developed for 6- and 12-month mortality outcome measures using backwards elimination with a fractional polynomial approach for continuous variables. Internal validation will be undertaken using bootstrapping methods. Discrimination of the model for 6- and 12-month mortality will be presented as receiver operating curves with c statistics. Calibration curves will be presented comparing observed and predicted event rates for each decile of risk as well as flexible calibration curves derived using loess-based functions.

**Discussion:**

The model developed in this study aims to improve clinical assessment of mortality risk for people with dementia living in RACFs in Australia. Further external validation in different populations will be required before the model could be developed into a tool to assist with clinical decision-making in the future.

## Background

Health professionals working in residential aged care facilities (RACFs) providing long-term care play a key role in end of life care decision-making for residents with dementia. Uncertainty regarding prognosis and difficulty identifying people with dementia whose health is deteriorating presents a significant barrier to initiating discussions of a palliative approach to care [1, 2]. In countries such as the USA, assessment of estimated life expectancy is a central consideration in determining who can access palliative care services such as hospice care [3] but health professionals often do not feel confident making prognostic assessments for people with dementia [4].

Planning for end of life care is infrequently discussed in the months prior to death for people with dementia living in RACFs [5] and emergency services are increasingly utilized toward the end of life [6]. In order to improve the quality of end of life care, timely recognition of end of life illness trajectory is essential to support carers and health care proxy decision-makers (usually the closest family member or a formally appointed decision-maker) of people with dementia in end of life care planning [7]. If health care proxy decision-makers are aware that end of life is approaching, people with advanced dementia are less likely to have invasive interventions in the months prior to death [8]. Lack of prognostic guidance is, however, a common barrier to health care proxy decision-makers engaging in decisions to avoid burdensome interventions such as hospital transfers [9].

There has been limited previous research aimed at identifying indicators of increased mortality risk for people with dementia living in RACFs. The Advanced Dementia Prognostic Tool (ADEPT) was developed to assess prognosis for residents with advanced dementia living in long-term care [10] but external prospective validation only demonstrated modest ability to discriminate for 6-month mortality (c statistic 0.67) [11]. The variables used to develop the ADEPT model were limited to those contained in the long-term care minimum data set (MDS), an administrative data set used throughout the USA for assessment of care quality and allocation of funding [12]. MDS data is not collected for the purpose of identifying indicators of deteriorating health for people with dementia and the accuracy of the clinical information contained within the MDS is variable [13]. Reliance on MDS data limits the clinical utility of the ADEPT model for use outside the US long-term care setting, and it is unknown whether there are clinical variables not collected in the MDS that would better identify people with dementia who are at increased risk of mortality.

In Australia, MDS assessments are not routinely undertaken and the Aged Care Funding Instrument (ACFI) is instead used to determine the level of care and funding [14]. ACFI assessments are infrequently updated following admission to residential care and in a recent cross-sectional analysis, the average time since last ACFI completion was greater than 12 months across RACFs in Australia [15], making these assessments unsuitable for detecting clinical deterioration. Important changes in clinical conditions not captured in ACFI data are recorded during routine clinical care but there is currently no standardized process for identifying and responding to prognostic indicators associated with increased mortality.

## Objectives

This study will aim to develop a prediction model for 6- and 12-month mortality for RACF residents with dementia using prognostic indicators recorded during routine clinical care. The model is intended to be applicable to people with dementia who have been living in a RACF for at least 3 months and to be utilized from 3 months onwards during the RACF admission.

## Methods

### Study design

This is a secondary analysis of a cohort of people with dementia from RACFs participating in a cluster-randomized trial of a simulation-based palliative care education intervention (IMPETUS-D) [16]. The cohort includes people with dementia living in RACFs in three states in Australia (Victoria, South Australia, and New South Wales) administered by a single-aged care provider. Residents do not receive any additional treatment or intervention through the IMPETUS-D program and participation is voluntary for all staff. Ethics approval for the study was obtained from the Melbourne Health Human Research and Ethics Committee (HREC/17/MH/336) as a component of the IMPETUS-D project ethics application. The study protocol has been designed in accordance with the TRIPOD statement for transparent reporting of the development of multivariable predictive models [17]. Prognostic indicator variables are extracted from RACF records retrospectively from the time of IMPETUS-D randomization using the date of most recent weight measurement as *t*0. Mortality will then be assessed prospectively for each resident at 6 and 12 months after *t*0.

### Data source

Data from RACF files has been accessed by site co-ordinators using a centralized database administered by the aged care provider. This database contains information recorded during routine clinical care and no assessment of residents will be undertaken outside of that required for usual care. Enrolment of participants occurred over a 12-month period during the pre-implementation and training phase of the IMPETUS-D project from December 2018, to December 2019, with baseline data and prognostic indicator variables collected at the time of enrolment. Mortality data will be collected until December 2020 and recorded at 6- and 12-month intervals post-assessment of prognostic indicator variables (*t*0). All study data will be recorded using the Research Electronic Data Capture tool (REDCAP) [18] in a secure online database administered by the University of Melbourne, Australia. The research database can only be accessed by research staff working on the project and all records will be stored for a minimum of 5 years after study completion.

### Participants

The cohort will comprise a minimum of 900 residents with dementia. Only residents who have been admitted to the RACF for a period of at least 90 days prior to enrolment will be eligible for inclusion if they have a documented diagnosis of dementia based on ICD-10 dementia definitions. Evidence of moderate to severe dementia will be required for study inclusion based on the cognitive skills assessment contained within the Aged Care Funding Instrument (ACFI) and dementia FAST staging.

The ACFI is a mandatory assessment instrument used to determine care needs and funding allocation for all residents of RACFs in Australia. Cognitive skills within the ACFI are divided into four groups (A, B, C, D) based on increasing degree of impairment such that group A has minimal impairment and group D has the most severe level of impairment. People with dementia with cognitive skills classified as group C or D (moderate to severe cognitive impairment) will be eligible for inclusion in the study. ACFI assessment of cognition uses the Psychogeriatric Assessment Scales—Cognitive Impairment Scale (PAS-CIS). This scale has been demonstrated to correlate well with the Mini-Mental State Examination (*r* = −0.77) [19] with higher scores indicating more severe impairment. Those with a PAS-CIS score of 10-15 are classified as cognitive skills C and those with a score of 16 or above as cognitive skills D. If the PAS-CIS score is not complete, the resident will be included if the ACFI appraisal determines that the resident meets the criteria to be classified as cognitive skills C or D based on evidence that they need assistance with the performance of activities of daily living due to cognitive impairment. In addition to ACFI criteria, study participants will need to meet the criteria for FAST stage 6e dementia at study entry. FAST staging correlates well with the trajectory of cognitive decline in moderate to advanced stages of dementia [20, 21] and stage 6e has been chosen as it represents the threshold for transition to the most advanced stage of dementia (FAST stage 7) for whom prognostic information may be particularly clinically relevant.

Baseline data collected will also include comorbid health conditions identified from diagnoses listed in RACF files matched to ICD-10 codes. Comorbidities will be clustered for analysis into the following groups: chronic lower respiratory diseases (J40-J47), chronic heart failure and cardiomyopathy (I11.0, I42, I50), chronic renal failure (N18-19), chronic liver disease (K70-K74), malignant neoplasms (C00-C97), and ischemic or hemorrhagic stroke (I60-I64).

The evolving Covid-19 (SARS-COV-2) pandemic [22] appears likely to impact RACFs in Australia during follow-up. Given the high mortality rate for Covid-19 among older adults and the unprecedented nature of the pandemic [23], participants who contract Covid-19 during follow-up will be excluded from the model development cohort.

### Outcomes

Mortality data will be collected prospectively through a review of RACF files to identify all residents who died during the follow-up period. For any study participants who leave the RACF prior to 12 months of follow-up mortality information will be requested from the Department of Births Deaths and Marriages. Mortality will be divided for analysis into those that occurred within 6 months of study entry and those that occurred within 12 months of study entry. These mortality end points were chosen as they were considered most clinically meaningful. Estimation of the likelihood of death within 6 months is required for hospice eligibility in the USA [24] and is the end point for which the ADEPT model was developed. The Australian Commission on Safety and Quality in Healthcare focuses on those who are “likely to die within the next 12 months” in guidelines regarding end of life care [25] and whether a clinician would be surprised by death within 12 months (“the surprise question”) is often used for end of life identification [26].

### Candidate predictor variables

The prognostic indicators for consideration in the multivariable model were identified from a literature review (unpublished) examining clinical features associated with increased mortality for people with dementia (see Table 1). Given the aim is to create a model that could be readily incorporated into routine clinical care, a parsimonious approach was taken to a selection of prognostic indicators with ten variables selected that could be easily assessed by RACF staff through a brief audit of the patient record. The number of variables chosen was also informed by consideration of the minimum number of events per variable required for model development (see sample size below).

**Table 1:** Candidate predictor variables

At study entry	Prior to study entry
Age [27]	Pressure injuries within the last 3 months [28, 29]
Sex [27]	Weight loss over the last 6 months [30]
Level of support required for oral intake [31]	Infections in the last 3 months [29]
Level of support required to transfer out of bed [31]	Hospital admissions in the last 3 months [32]
BMI [33]	Number of falls in the last 3 months [34]

Pressure injuries, infections, falls, and hospital admissions are recorded through mandatory clinical documentation during routine care at the participating RACF sites. The frequency of these clinical episodes will be assessed for 3 months prior to study entry. More than 10% weight loss in 6 months has been identified as a predictor of mortality in previous studies and thus a 6-month period was chosen for calculation of percentage weight loss [10, 30].

The level of staff assistance for oral intake and transferring out of bed will be assessed from care documented in RACF files and quantified using descriptors from the Minimum Data Set ADL Scale (MDS ADL). The MDS ADL scale has been validated against other functional assessment scales in long-term care [35] and is able to detect change in function for people with cognitive impairment living in RACFs [36]. A decline in MDS ADL scale scores for oral intake and transferring ability, in particular, occurs more commonly in the last months of life for people with advanced dementia [31]. The MDS ADL scale divides the level of assistance required to complete a task into five categories of increasing dependence. A score of zero is given for independence, increasing to a score of four for total dependence on staff to complete the activity.

### Sample size

The study cohort will be derived from 24 RACFs comprising a minimum of 2400 residents receiving long-term care. Assuming that 50% of residents will have a diagnosis of dementia based on previous published data from long-term care in Australia [37], there will be an estimated minimum of 1200 residents with dementia. Three quarters (77.3%) of people with dementia in RACFs in Australia have moderate to severe cognitive impairment [38] equating to a minimum of 900 people with dementia who would meet the criteria for inclusion in the cohort.

A minimum of 10 events per variable has been proposed as a reasonable sample size for the development of predictive models [39] and increasing the sample size to 20 events per variable appears to improve the reliability of results [40]. For the ten prognostic indicator variables, a minimum of 200 events (deaths) are therefore required to reach a minimum of 20 events per variable. In RACFs in Australia, the 12-month mortality rate for people with dementia is approximately 30% [37], which is similar to mortality rates found in cohort studies of people with dementia in long-term care in the USA [41, 42]. Assuming a mortality rate of 30%, the required minimum cohort size for the model development cohort is therefore 667 people with dementia to observe a minimum of 200 deaths.

An alternative method for estimating the minimum sample size required for model development has been recently proposed by Riley et al. [43]. Using the optimism corrected c statistic for 12-month mortality reported for the ADEPT model (*c* = 0.68) [42], a *R*^2^_D_ value of 0.208 can be derived. This value can then be used to estimate sample size when combined with a prespecified level of shrinkage for the final model along with the expected event rate and the number of candidate predictor variables. Using the pmsampsize package in R version 3.6.2 with an *R*^2^ of 0.208, shrinkage factor of 0.9, event rate of 0.3, and 10 predictor variables gives a minimum sample size of 381 with 11.43 events per variable. The estimated minimum study cohort of 900 people will therefore exceed this figure and also the more conservative 20 events per variable estimate.

### Missing data

Missing data will be reported for each variable and addressed through multiple imputation using chained equations (MICE) [44]. Assuming that data is missing at random, multiple imputation allows analysis of variables with missing data and is preferable to restricting analysis to only include those with complete data [45, 46]. The number of imputations will be determined based on the fraction of missing information (FMI) such that the number of imputations undertaken equals the percentage value of the FMI for example using 20 imputations if the FMI is 0.2 [44]. When the FMI is greater than 0.5, this relationship does not hold and greater than 100 imputations may be needed [47]. Given the uncertainty with multiple imputation when the FMI is greater than 0.5, if greater than 50% of cases having missing data, a complete case analysis will be undertaken for sensitivity analysis.

### Statistical analysis

Multivariable logistic regression will be used for model development rather than time-to-event methods such as the cox proportional hazards model. We expect to have complete outcome data for all participants at 12 months and have chosen the end points of 6- and 12-months for model development as these have particular clinical relevance. Using logistic regression to model these outcomes does not require an assumption of proportional hazards that may not apply to the prognostic indicators included in the model. The model will be developed through backwards elimination with a nominal level of alpha for variable exclusion set at 0.1 (10%). Using overly stringent levels of alpha for backwards elimination such as 0.05 is more likely to create an overly simplified model that eliminates relevant prognostic variables [48]. Non-linearity of continuous variables will be addressed by using a multivariable fractional polynomial approach, an established technique for transforming non-linear continuous variables when developing a backwards elimination model [49, 50]. The effect of exposure to the IMPETUS-D palliative care education intervention will be analyzed as an additional variable in multivariable analysis to assess whether the intervention influenced mortality. This approach does not require exclusion of those who received the intervention from model development reducing the potential for over-fitting of the model which is more likely to occur if model development is limited to the control group only [51]. A sensitivity analysis of model performance in both the intervention and control groups will also be undertaken.

Performance of the model will be assessed through calculating c statistics and receiver operating curves describing model discrimination for 6 and 12 months mortality. Internal validation will be undertaken using bootstrapping resampling methods [52], which account for bias due to over-fitting more accurately than split sample cross-validation approaches [53]. The model development process will be repeated in 400 bootstrap samples to allow calculation of optimism adjusted calibration slopes and c statistics. Internal calibration will be assessed through comparing observed and predicted event rates for each decile of risk predicted by the model and plotting flexible calibration curves using loess-based smoothers [54]. This approach is considered more informative than the Hosmer-Lemeshow test regarding potential miscalibration [55, 56].

In addition to the model building process, a narrative analysis will also be presented describing the cohort at baseline with regard to age, gender, time since admission to residential care, level of functional impairment on MDS-ADL scale, and comorbidity burden. The spread of mortality rates across RACFs involved in the study will also be examined to explore heterogeneity between facilities. To examine heterogeneity between study sites further, a leave-center-out cross-validation approach will be applied using each RACF as an individual center (*n* = 24) to allow analysis of variability in the developed model in settings of differing baseline mortality risk.

### Reporting of results

Results will be reported in accordance with the Tripod checklist for reporting studies developing multivariable predictive models [17]. The flow of participants through the study will be presented along with their baseline characteristics and the number of participants with missing data. The distribution of participants between intervention and control arms of the IMPETUS-D palliative care education trial will be reported and analysis of the effect of exposure to the intervention on mortality outcomes will be reported. The unadjusted association between each predictor variable and mortality outcomes will be presented in addition to the results of the multivariable analysis and details of how the final prediction model was developed.

## Discussion

This study aims to develop a mortality prediction model for people with dementia using prognostic indicators assessed during routine care provided in Australian RACFs. Rather than previous models that have been developed from minimum data set (MDS) variables in the USA, this model will be developed from predefined prognostic indicator variables that may better identify people with dementia at an increased risk of mortality. We believe the inclusion of variables that identify features of recent health instability such as infections, falls, and hospitalizations will identify people with dementia whose health is deteriorating better than a model that only includes factors describing health status at the time of assessment.

One of the limitations of this study is that prognostic indicator variables will be collected retrospectively from RACF files and there is a risk of under detection of variables if documentation is incomplete. While relying on routinely documented clinical information as the source of prognostic information has limitations, this approach reflects how the model would likely be used in clinical practice. A model that requires additional prospective assessment of residents outside of usual care is unlikely to be implemented in the RACF care environment in Australia given that uptake of prospective assessment tools for aspects of dementia care such as pain and behavioral assessment has been very limited unless mandated for use [57, 58].

The developed model should be considered preliminary and not be incorporated into clinical care without further study. In the current study design, heterogeneity in the duration of time the cohort has been living in long-term care at study entry (*t*0) may introduce survivorship bias. People with dementia newly admitted to long-term care have higher mortality rates within the first 3 months [42, 59] and excluding this group aims to develop a model that only applies to survivors of this high-risk period. To address the issue of survivorship bias, further study is needed involving a cohort followed prospectively from 3 months after RACF admission. Future research combining serial assessment of prognostic indicator variables with dynamic modeling techniques would also allow the model to be utilized across multiple time points. Comparing the performance of the developed model to the ADEPT model in an Australian RACF cohort will also be an important aspect of further validation of the model. The variables required for the ADEPT model are not routinely assessed during clinical care at RACFs in Australia and require additional assessment of residents that is beyond the scope of this model development study.

Even after further validation, there will always be uncertainty with predictive models and the aim is to provide guidance about potential illness trajectory to health care providers rather than replace clinical judgment. It is hoped that, once externally validated, a clinical tool could be developed to enable better identification of residents with dementia at greatest risk of mortality over the following 12 months for whom access to resources such as specialist geriatric medicine and palliative care support may be most critically needed to optimize care and facilitate anticipatory end of life care planning. Future development of a decision support tool would require the involvement of health professionals working in RACFs, people with dementia, and their caregiver’s in order to ensure that mortality risk information is utilized appropriately to meet the needs of the population that the tool intends to support.

## Data Availability

Not applicable.
