# Correction to: A study protocol for the development of a multivariable model predicting 6- and 12-month mortality for people with dementia living in residential aged care facilities (RACFs) in Australia

**DOI:** 10.1186/s41512-021-00099-2

**Published:** 2021-04-16

**Authors:** Ross Bicknell, Wen Kwang Lim, Andrea B. Maier, Dina LoGiudice

**Affiliations:** 1 Department of Medicine and Aged Care, @AgeMelbourne, Melbourne Health–Royal Melbourne Hospital, University of Melbourne, 6 North Main Building, Royal Melbourne Hospital, 300 Grattan Street, Parkville, Victoria 3050 Australia; 2 Department of Human Movement Sciences, @AgeAmsterdam, Amsterdam Movement Sciences, Vrije Universiteit Amsterdam, Amsterdam, The Netherlands

**Correction to: Diagn Progn Res 4, 17 (2020)**

**https://doi.org/10.1186/s41512-020-00085-0**

Following publication of the original article [1], the authors identified an error in the author name of Dina LoGiudice.

The incorrect author name is: Dina LoGiuidice.

The correct author name is: Dina LoGiudice.

The author group has been updated above and the original article [1] has been corrected.
